# Detection and Tracking of Moving Targets for Thermal Infrared Video Sequences

**DOI:** 10.3390/s18113944

**Published:** 2018-11-14

**Authors:** Chenming Li, Wenguang Wang

**Affiliations:** School of Electronic and Information Engineering, Beihang University, Beijing 100191, China; lchm1990@163.com

**Keywords:** joint detection and tracking of multi-target, thermal infrared (TIR) image, track-before-detect (TBD), background subtraction, labeled random finite sets (RFS), *δ*-GLMB filter

## Abstract

The joint detection and tracking of multiple targets from raw thermal infrared (TIR) image observations plays a significant role in the video surveillance field, and it has extensive applied foreground and practical value. In this paper, a novel multiple-target track-before-detect (TBD) method, which is based on background subtraction within the framework of labeled random finite sets (RFS) is presented. First, a background subtraction method based on a random selection strategy is exploited to obtain the foreground probability map from a TIR sequence. Second, in the foreground probability map, the probability of each pixel belonging to a target is calculated by non-overlapping multi-target likelihood. Finally, a δ generalized labeled multi-Bernoulli (δ-GLMB) filter is employed to produce the states of multi-target along with their labels. Unlike other RFS-based filters, the proposed approach describes the target state by a pixel set instead of a single point. To meet the requirement of factual application, some extra procedures, including pixel sampling and update, target merging and splitting, and new birth target initialization, are incorporated into the algorithm. The experimental results show that the proposed method performs better in multi-target detection than six compared methods. Also, the method is effective for the continuous tracking of multi-targets.

## 1. Introduction

The detection and tracking of moving targets is a challenging vision task that has attracted extensive research. Because of the comparatively lower cost, omnipresence, and 24 × 7 applicability, thermal infrared (TIR) sensors have provided new application areas [1]. Since pedestrians are the major participants in many events of interest, the joint detection and tracking of multi-targets (usually meaning pedestrians, but not exclusively in this paper) becomes one primary task borne by the TIR surveillance system [2]. The main advantages of thermal sensors are their ability to see in complete darkness, their robustness to illumination changes and shadow effects, and their comparatively lower degree of intrusion regarding privacy. Despite many superiorities, the main disadvantages of TIR imaging include low resolution, many dead pixels, lack of color information, low foreground/background contrast, and associated heavy noise [2,3]. As well as the above disadvantages, in top-down surveillance scenes, the target usually occupies fewer pixels and it is difficult to extract the effective appearance features, including textural and contour information. Moreover, the targets in surveillance scenes are highly variable in pose, size, shape, and intensity [2]. Multiple moving-target detection and tracking remain crucial objectives and are identified as the key issues in the TIR surveillance system.

Usually, detection and tracking tasks are separable in computer vision, and most multi-target tracking objectives require a detection operation to produce measurements [4]. Emerging technologies, such as proposal detection method and deep convolution neural network method, usually cannot achieve as admirable detection performance in the TIR surveillance images as the optical images due to the above-mentioned shortcomings. The traditional method still plays a significant role in moving-target detection. In addition, the detection methods in video streams can be divided into three categories: frame difference, optical flow, and background subtraction methods [5]. Because of its low computational cost and high accuracy, the background subtraction method is more popular [6,7]. Unfortunately, the classic background subtraction method has two debilitating drawbacks. First, it achieves detection tasks based on a threshold, which results in information loss [8]. During detection, information loss can significantly degrade tracking performance, especially in obscure feature cases [4,9]. Second, the detection foreground pixels from different targets are not discriminable. To track different targets individually, some post-processes must be executed to differentiate them. Therefore, the joint processing of detection and tracking tasks is of fundamental interest to reduce information loss and simplify the process. Some typical algorithms such as dynamic programming [9,10], Bayesian existence process [11], and multi-modal distributions [12] have shown great success. Among them, the random finite set (RFS) framework approaches that jointly detect and track have attracted significant attention [4,13,14].

The RFS-based methods consist of two procedures in a Bayesian framework: prediction and update. Two methods can be used to implement them, one based on the Gaussian mixtures (GM) model and the other based on the sequential Monte Carlo (SMC) model; the latter is also known as particle implementation. The RFS-based filters can be divided into unlabeled filters and labeled filters. The probability hypothesis density (PHD), cardinalized PHD (CPHD), and multi-target multi-Bernoulli (MeMBer) filters are the typical unlabeled category [15,16,17]. Although these filters have been successfully applied to visual tracking [4,18,19], they provide only unidentified estimates and require additional post-processing to form tracks. The generalized labeled multi-Bernoulli (GLMB) filter and δ-GLMB filter belonging to the label class of RFS-based filters can distinguish and maintain different tracks by adding a label to each target [20,21]. Based on the labeled RFS filter, a δ-GLMB track-before-detect (TBD) approach with a separable likelihood function was introduced in a radar-tracking scenario in [22]. Subsequently, an improved GLMB-TBD algorithm, which can handle non-separable likelihood situations, was proposed in [23] for generic measurement models, although the considerable computational cost limits its application. Currently, the labeled RFS-based methods focus on non-overlapping targets and point target tracking [14,18,22].

To alleviate the above problem in multi-target detection and tracking of TIR surveillance system, a joint detection and tracking approach based on particle implementation, which combines a background subtraction method with a GLMB filter, is proposed. First, according to the ViBe algorithm [24], a random selection strategy background subtraction method without threshold detection is designed to yield a TIR foreground probability map. Second, a multi-target likelihood function is used to calculate the probability of each pixel belonging to a target in the foreground probability map. Finally, the δ-GLMB-TBD filter is applied to produce the state of the multi-target along with their labels. Most RFS-based TBD methods describe a target in the image as a rectangle or a single point [14,18], which is too rough for a target with complex contour and may degrade the detection and tracking performance. In the proposed method, the target is represented by its own pixel set. This means that the δ-GLMB-TBD filter is extended to track irregular areas of a target by benefiting from shape similarity in consecutive frames. To be practical, the algorithm also includes some extra procedures, such as pixel sampling and update, target merging and splitting, and new birth target initialization, which accommodate target deformation and overlapped and dynamic change via gathering, splitting, birth, and death.

The main contribution of this paper is the proposal of a joint multi-target detection and tracking method based on background subtraction and a δ-GLMB-TBD filter for infrared surveillance system, along with its particle implementation. As well as producing a multi-target state estimate, the proposed method can track the multi-targets successfully and individually keep their labels. Based on the proposed method, we also developed the following:

- More effective multi-target estimates which are from a pixel set instead of a rectangle or a single point;
- Several procedures to accommodate target deformation and multi-target dynamic processes, such as pixel sampling and update, target merging, splitting, and new target initialization;
- A random selection strategy background subtraction method which can be used to pre-process the images without threshold segmentation.

This paper is organized as follows. Section 2 describes the proposed algorithm in detail, including the background subtraction method, multi-target likelihood calculation, and the recursion of the δ-GLMB-TBD filter. The results and analysis of the experiments are presented in Section 3. Conclusions are drawn in Section 4.

## 2. Background-Subtraction-Based δ-GLMB-TBD Filter

**Figure 1:**
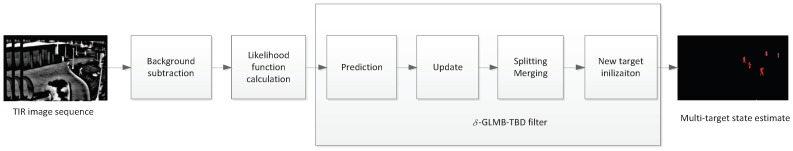
Schematic of the proposed method.

In this section, the background-subtraction-based δ-GLMB-TBD filter is introduced in three major parts: background subtraction, multi-target likelihood function calculation, and implementation of the δ-GLMB-TBD filter [22]. A block diagram is presented in Figure 1. Background subtraction transforms the original TIR image to a foreground probability map where each pixel can be interpreted as the probability of the pixel belonging to the foreground. Then the map is used to generate new birth targets and to calculate the multi-target likelihood. Finally, the δ-GLMB-TBD is used to produce multi-target estimates. To accommodate target deformation and dynamic change, some extra procedures, such as splitting and merging, are included.

### 2.1. Background Subtraction Method for TIR

**Figure 2:**
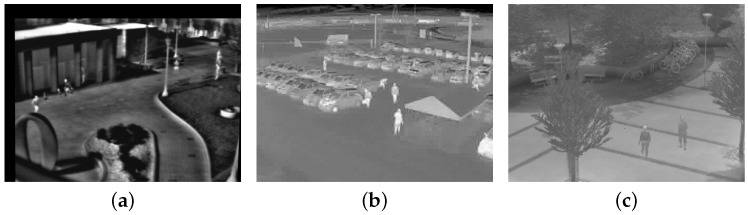
Three typical TIR images. (**a**) Campus; (**b**) Parking lot; (**c**) Community.

Figure 2 shows three typical TIR surveillance scenes, in which it is difficult to detect all targets because of low foreground/background contrast, fewer pixels, heavy noise, and lack of textural and contour information. One popular method is background subtraction. However, background subtraction usually produces two segmentations that denote background and foreground by threshold detection, which results in information loss and then leads to inferior tracking performance. To alleviate these problems, the GLMB-TBD filter without the need to detect targets is employed to achieve the joint detection and tracking of multi-target on a background suppression image. Many methods have the potential to subtract the background, such as ViBe [24], KDE [25], GMM [26] and so on. In this paper, we propose a random selection strategy based on ViBe algorithm [24] to subtract the background, which is because the ViBe algorithm has the characters of low complexity, high stability, and excellent background subtraction effect [27]. The new background subtraction method consists of two parts: background model initialization and background model update. The differences between the new approach and the ViBe method are as follows: (1) less missing detection without threshold segmentation; (2) pixel background model update with respect to the probability of being a foreground pixel; (3) morphology operations to eliminate scattered noise and maintain shape. The details are introduced in the following sections.

#### 2.1.1. ViBe Method

Before discussing the proposed method, we will first summarize the standard ViBe method. The background subtraction is regarded as a classification problem in the ViBe method. However, as there is no way to model each background pixel as a probability density function (PDF), then, no estimation or classification result could be given directly from the PDF. Therefore, the ViBe method establishes a background pixel set for each pixel, and each element in this set can be seen as a sample obtained by the true PDF of the background [24].

The key problems of the ViBe method are: (1) how to get the background pixel set, effectively; and (2) how to classify a pixel as a background or foreground according to its given background pixel set. For the first problem, a random selection strategy-based method is employed to update the background pixel set. This can make the samples be more compliant to the pdf of the background without increasing the number of the samples and discarding the earlier samples. For the second problem, the new pixel should compare with its background pixel set. The steps are listed as follows.

Step 1: Calculate the Euclidean distances between the new sample and each sample in its background pixel set;

Step 2: Obtain the number of the Euclidean distances shorter than a given threshold;

Step 3: Compare the number with another given threshold; if the number is greater than the threshold, then the new pixel is classified as a background pixel and vice versa.

#### 2.1.2. Background Model Initialization

Regarding the proposed method, the background model initialization will be discussed first. The proposed method only employs the first frame to initialize the background model. Let $y_{i}$ denote the ith pixel value in the original TIR image $I_{ori}$ ($1\leq i\leq N_{img}$ , where $N_{img}$ denotes the number of all pixels in the TIR image), and $M_{i}\left(k\right)$ denotes the background model of pixel *i* at time *k* (in the background model initialization, set k=1); all $M_{i}\left(k\right)$ make up the image background model M(k). Each $M_{i}\left(k\right)$ is a collection of *N* background samples.

(1) $$M_{i}\left(k\right)=\left\{m_{i,1},m_{i,2},...,m_{i,N}\right\}.$$

where $m_{i,n}$ (1≤n≤N) is a sample and initialized as follows:

(2) $$m_{i,n}=y_{i}+v_{ran}\left(v_{l},v_{h}\right)$$

where $v_{ran}\left(v_{l},v_{h}\right)$ denotes a uniform random number between $v_{l}$ and $v_{h}$. The main parameter in initial model is: *N*.

#### 2.1.3. Background Model Update

The background model will be updated with each new frame. The update step is the core procedure used to yield accurate results over time. In this step, a conservative update policy is used.

For each pixel *i* in frame $I_{ori}$ at time *k* (k>1), its equivalent background is the average of its background model $M_{i}\left(k\right)$ given by

(3) $$y_{i,equ}=\frac{1}{N}\left(m_{i,1}+m_{i,2}+...+m_{i,N}\right).$$

We compare the absolute difference between its current value $y_{i}$ and its equivalent $y_{i.equ}$ with a threshold $th_{diff}$. The comparison result $s_{i}$ can be obtained by

(4) $$s_{i}=\left\{\begin{array}{cc} abs\left(y_{i}-y_{i,equ}\right)/th_{diff} & if\;\;abs\left(y_{i}-y_{i,equ}\right)<th_{diff}\\ 1 & if\;\;abs\left(y_{i}-y_{i,equ}\right)\geq th_{diff} \end{array}\right.$$

where abs(·) denotes the “absolute” operation. $s_{i}$ = 1 means the pixel *i* is classified as foreground. According to a conservative update policy, a foreground pixel should never be used to update the background. Thus, only when $s_{i}<1$ can the current pixel value $y_{i}$ replace one sample in the background model $M_{i}\left(k-1\right)$ with the probability of ($1-s_{i}$). So $th_{diff}$ plays a significant role in updating the background model. When $th_{diff}$ is too small, $s_{i}$ is sensitive to the pixel change and noise; when $th_{diff}$ is too large, the background model may be polluted by the pixel belonging to moving target. In contrast with the first-in-first-out strategy, the sample substituted by $y_{i}$ is chosen randomly by a uniform probability density function. These operations can extend the time windows covered by the background models. The long lifespan of the background samples significantly aids in the detection of slow-moving multi-targets. The conservative update policy can make a sharp detection of a moving target without introducing the foreground.

Unfortunately, one disadvantage of the conservative update policy is that it can lead to deadlock situations and ghosts. To eliminate these influences, an improved “detection support map” method [28], which counts the number of times a pixel is classified as foreground consecutively, is employed. At frame *k*, the detection support map $DSM_{i}\left(k\right)$ is given by

(5) $$DSM_{i}\left(k\right)=\left\{\begin{array}{cc} DSM_{i}\left(k-1\right)+1 & s_{i}=1\\ 0 & otherwise \end{array}\right.$$

We assume the maximum duration of a target remaining stationary is $t_{sta}$ and that the corresponding frame number is $N_{sta}$. If the time of one target remaining stationary reaches $t_{sta}$, this target is then classified as a background target. When the $DSM_{i}\left(k\right)$ reaches the threshold $N_{sta}$, a random strategy is then used to update the background model. During the update, $n_{ran}$ samples ($n_{ran}$ is a uniform random positive integer between 1 and *N*) in the background model of pixel *i* are replaced by new samples. To speed up ghost elimination, when a sample has been updated, one sample from its 8-connected neighborhood pixel background model should be replaced by a new value according to a uniform law. This operation uses the spatial consistency assumption of the background, as in the ViBe method. The assumption is that the background pixel shares a similar distribution as its immediate neighbors. In addition, each new sample is obtained by (2). The detection support map is updated following (6), and the updated background model $M_{i}\left(k\right)$ at time *k* is obtained.

(6) $$DSM_{i}\left(k\right)=DSM_{i}\left(k\right)-n_{ran}$$

**Figure 3:**
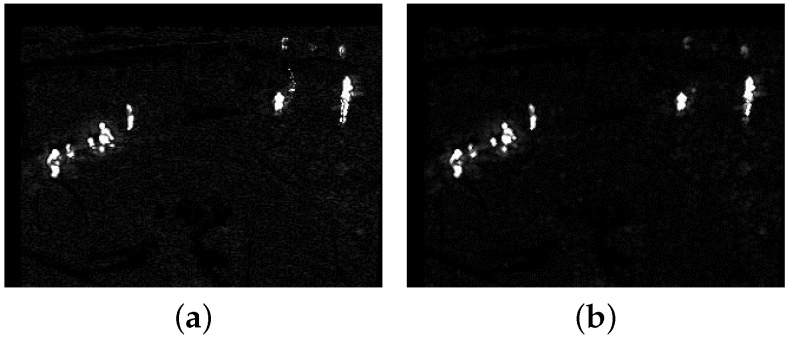
The two foreground probability maps of Figure 2a. (**a**) The original foreground probability map; (**b**) the foreground probability map with dilation following erosion.

Based on the above steps, the whole update background model M(k) can be obtained by recursion with the new TIR frame $I_{ori}$. Then, (4) can be executed with the updated $y_{i,equ}$ calculated from $M_{i}\left(k\right)$; all $s_{i}$ ($1\leq i\leq N_{img}$) constitute the foreground probability map S(k). Figure 3a shows the foreground probability map of Figure 2a. The whole target is separated into several small targets and there is some scattered noise in S(k) in Figure 3a. Morphology erosion with small structure can be used to eliminate the scattered noise. Before normalizing the absolute difference by $th_{diff}$ in (4), we conduct erosion and dilation operation on the absolute difference image whose pixel value is $abs\left(y_{i}-y_{i,equ}\right)$. Figure 3b shows the foreground probability map with dilation following erosion. The pseudo-code for the background model update is presented in Algorithm 1. Lines 1–3 determine a pixel to be a foreground pixel ($s_{i}=1$) or a background pixel ($s_{i}<1$). If the pixel belongs to the background, then we update its background model as lines 4-9. If it belongs to the foreground, besides updating $M_{i}\left(k-1\right)$ lines 11–18 also show how to use the detection support map to eliminate deadlock situations and ghosts.

**Algorithm 1:** Background model update

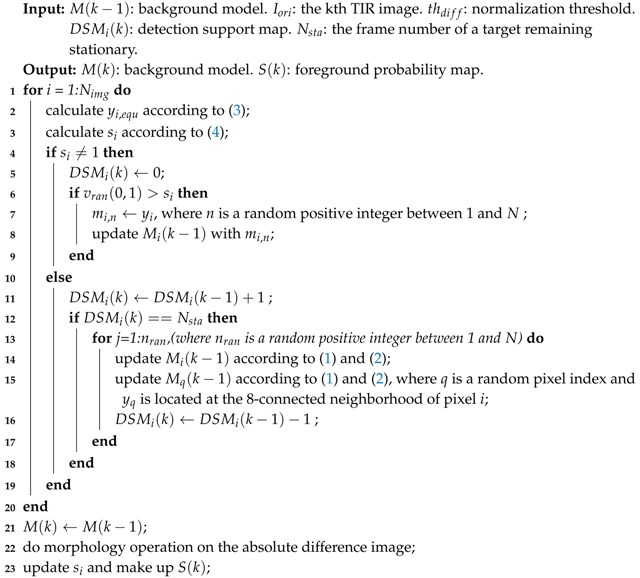



### 2.2. Multi-Target Likelihood Function Calculation

In the foreground probability map S(k), each $s_{i}$ ($1\leq i\leq N_{img}$) lies in the interval [0, 1], which can be interpreted as the probability that pixel *i* should be classified as the foreground. In S(k), each target *x* illuminates a set of pixels denoted by T(x). Inspired by [4], if a pixel i∈T(x), its intensity distribution follows the foreground likelihood function $g_{F}\left(x\right)$, and if i∉T(x), its intensity distribution follows the background likelihood function $g_{B}\left(x\right)$. These two likelihood functions (or probability density functions of intensity) are of the form:

(7) gF(x)=ςFexp(xxδFδF)

(8) gB(x)=ςBexp(-xxδBδB)

where $\delta_{F}$ and $\delta_{B}$ determine the spread rates of the foreground and background intensities respectively, and $\varsigma_{F}$ and $\varsigma_{B}$ are normalizing factors. In general, the ratio of $\delta_{F}$ to $\delta_{B}$ determines the detection threshold for judging whether a pixel belongs to a target or background. The greater the ratio is, the lower the associated target threshold will be. Because the TBD method allows more suspected targets to be input into the δ-GLMB-TBD filter, $\delta_{B}$ should be significantly smaller than $\delta_{F}$. The background intensity remains constant unless it is quite close to the target, whereas the foreground intensity has a significantly more variable and spreading intensity function [4,29]. As the references state, the fluctuation of the ratio does not cause significant changes to the tracking results.

Let $\bar{s}\left(x\right)$ denote the average of all pixels in T(x), i.e.,

(9) $$\bar{s}\left(x\right)=\frac{1}{\left|T(x)\right|}\sum_{i\in T(x)}s_{i}$$

where $\left|\cdot \right|$ denotes the cardinality (the number of elements) of a set. We assume that all illumination regions of influences of the multi-target in the TIR image are not overlapped, i.e., $x\neq x^{\prime }\Rightarrow T\left(x\right)\cap T\left(x^{\prime }\right)=\emptyset$. This assumption is reasonable, because an individual cannot recognize the identities and states of overlapped targets. For example, in the TIR image when some pedestrians overlap with each other, an individual cannot determine whether one of the pedestrians has disappeared, whether a pedestrian appears in the surveillance scene, or when or whether the group of pedestrians will separate from each other. In practical application, tracking is a dynamic process. When targets are overlapped, the merging procedure (seen in Section 2.3) will guarantee that all targets are treated as one. After separation, they will be tracked individually by the splitting procedure (seen in Section 2.3). The GLMB-TBD filter will generate the separable likelihood function based on the assumption that the targets do not overlap. Then, if given a target set *X* with statistically independent pixel values, the likelihood that the set *X* illuminates the region ${⋃}_{x\in X}T(x)$ can be expressed as $\prod_{x\in X}g_{F}\left(\bar{s}\left(x\right)\right)$.

Let ${\bar{s}}_{B}\left(X\right)$ denote the average intensity of the map S(k) after filling all target regions with the background pixel value of 0, i.e.,

(10) $${\bar{s}}_{B}\left(X\right)=\frac{1}{N_{img}}\left(\sum_{i=1}^{N_{img}}s_{i}-\sum_{x\in X}\sum_{i\in T(x)}s_{i}\right).$$

Substituting ${\bar{s}}_{B}\left(X\right)$ into (8),

(11) $$\begin{array}{c} g_{B}\left({\bar{s}}_{B}\left(X\right)\right)=\varsigma_{B}\exp\left(-\frac{\sum_{i=1}^{N_{img}}s_{i}-\sum_{x\in X}\sum_{i\in T(x)}s_{i}}{\delta_{B}N_{img}}\right)\\ =\varsigma_{B}\exp\left(-\frac{\sum_{i=1}^{N_{img}}s_{i}}{\delta_{B}N_{img}}\right)\prod_{x\in X}\exp\left(\frac{\sum_{i\in T(x)}s_{i}}{\delta_{B}N_{img}}\right)\\ =\varsigma_{B}\exp\left(-\frac{\sum_{i=1}^{N_{img}}s_{i}}{\delta_{B}N_{img}}\right)\prod_{x\in X}\exp\left(\frac{\left|T(x)\right|\cdot \bar{s}\left(x\right)}{\delta_{B}N_{img}}\right). \end{array}$$

Then, given the target set *X*, the multi-target likelihood of S(k) is the product of the foreground and background, i.e.,

(12) $$\begin{array}{c} g\left(S\left(k\right)|X\right)=g_{B}\left({\bar{s}}_{B}\left(X\right)\right)\prod_{x\in X}g_{F}\left(\bar{s}\left(x\right)\right)\\ =\underset{independent\;of\;X}{\underset{︸}{\varsigma_{B}\exp\left(-\frac{\sum_{i=1}^{N_{img}}s_{i}}{\delta_{B}N_{img}}\right)}}\prod_{x\in X}\underset{dependent\;on\;x}{\underset{︸}{\exp\left(\frac{\left|T(x)\right|\cdot \bar{s}\left(x\right)}{\delta_{B}N_{img}}\right)g_{F}\left(\bar{s}\left(x\right)\right)}}. \end{array}$$

(12) shows that this likelihood is separable.

### 2.3. δ-GLMB-TBD Filter

After obtaining the multi-target likelihood for the foreground probability map, we describe how to detect and track multi-targets using the improved δ-GLMB-TBD filter with a separable likelihood function [30]. The main contribution in this part is that the improved filter can produce the appearance of the target in contrast to the center position or rectangle estimates obtained by the standard δ-GLMB-TBD filter. Based on this, we also develop the following: (1) merging and splitting procedures are employed to handle situations where multi-targets merge into one group and one group splits into several multi-targets; (2) pixel sampling and updating are used to accommodate target deformation; (3) birth target initialization procedure is to open up the applications of the filter. We discuss each of these improvements in the following subsections.

#### 2.3.1. Basic Theory

In this subsection, the standard δ-GLMB-TBD filter, consisting of two steps, prediction and update, with a separable likelihood function, is briefly discussed [23]. Before introducing the recursion of the standard δ-GLMB-TBD filter, some notations are shown for convenience.

(1) Notation 

For the remainder of the paper, let lowercase letters (e.g., *x*) denote single-target state and uppercase letters (e.g., *X*) denote multi-target states. The labeled target states are indicated by boldface letters (e.g., x, X). Space is represented by a letter with a tilde (e.g., $\tilde{X}$ denotes the state space, $\tilde{L}$ denotes the discrete label space). A labeled target can be written as x=(x,l), where $l\in \tilde{L}$ and l=(k,i), *k* means the target birth time, and *i* is a unique index to distinguish targets born at the same time. A labeled multi-Bernoulli (LMB) RFS with state space $\tilde{X}$ and label space $\tilde{L}$ can be written as $v={\left\{r^{\left(l\right)},p^{\left(l\right)}\right\}}_{l\in \tilde{L}}$, where $r^{\left(l\right)}$ and $p^{\left(l\right)}$ mean the existence probability and the probability density of a target with label *l*. $\langle \alpha ,\beta \rangle =\int \alpha (x)\beta (x)dx$ denotes the standard inner product, and $h^{X}=\prod_{x\in X}h(x)$ denotes the multi-target exponential, where $h^{\emptyset }=1$ by convention and α(x), β(x), and h(x) are real-valued functions. The generalized Kronecker delta function and inclusion function are defined as

(13) $$\delta_{Y}\left(X\right)=\left\{\begin{array}{cc} 1 & if\;X=Y\\ 0 & otherwise \end{array}\right.,$$

(14) $$1_{Y}\left(X\right)=\left\{\begin{array}{cc} 1 & if\;X\subseteq Y\\ 0 & otherwise \end{array}\right..$$

(2) Standard δ-GLMB-TBD Filter 

From [23], the multi-target posterior at time *k* has the following δ-GLMB form:

(15) $$v_{k}\left(X\right)=\Delta \left(X\right)\sum_{I\in F({\tilde{L}}_{0:k})}\delta_{I}\left(L\left(X\right)\right)w_{k}^{\left(I\right)}{\left[p_{k}^{\left(I\right)}\right]}^{X}$$

where X is the current multi-target state; $\Delta \left(X\right)=\delta_{\left|X\right|}\left(\left|L(X)\right|\right)$ denotes the distinct label indicator, which means that the cardinalities of the set of labels and the set of state vectors are identical; ${\tilde{L}}_{0:k}$ denotes the label space of targets born between time 0 and time *k*, where the subscript 0:k means time interval [0,k]; F(·) denotes collections of all finite subsets of a given space; L is a projection from space $\tilde{X}\times \tilde{L}$ to $\tilde{L}$ and hence L(X)={L(x):x∈X} is the set of labels of X; and $w_{k}^{\left(I\right)}$ denotes the joint existence probability of the label set *I*, while the multi-target exponential ${\left[p_{k}^{\left(I\right)}\right]}^{X}$ denotes the joint probability density of X, conditional on their corresponding label set *I*.

The new birth model covering labeled Poisson, labeled identically and independently distributed cluster and labeled multi-Bernoulli filter can be given by [20]

(16) $$f_{B}=\Delta \left(Y\right)w_{B}\left(L\left(Y\right)\right){\left[p_{B}\right]}^{Y}.$$

where Y is the state of new birth targets, and $w_{B}$ and ${\left[p_{B}\right]}^{Y}$ are the joint existence probability and probability density. This model can also be written as the LMB birth model [23,31] as follows:

(17) $$w_{B}\left(L\right)=\prod_{i\in {\tilde{L}}_{k}}\left(1-r_{B}^{\left(i\right)}\right)\prod_{l\in L}\frac{1_{{\tilde{L}}_{k}}\left(l\right)r_{B}^{\left(l\right)}}{1-r_{B}^{\left(l\right)}}$$

(18) $$p_{B}\left(x,l\right)=p_{B}^{\left(l\right)}\left(x\right)$$

where $r_{B}^{\left(l\right)}$ and $p_{B}^{\left(l\right)}\left(x\right)$ mean the existence probability and the probability density of a birth target with label *l*.

**Proposition** **1.** *If the multi-target state posterior with δ-GLMB form is given as (15) at time k, with the new birth model (16), the multi-target prediction density also has a δ-GLMB form [23]:*

(19) $$v_{k+1|k}\left(X\right)=\Delta \left(X\right)\sum_{I\in F({\tilde{L}}_{0:k+1})}\delta_{I}\left(L\left(X\right)\right)w_{k+1|k}^{\left(I\right)}{\left[p_{k+1|k}^{\left(I\right)}\right]}^{X}$$

*where*

(20) $$w_{k+1|k}^{\left(I\right)}=w_{S}^{\left(I\right)}\left(I\cap {\tilde{L}}_{0:k}\right)w_{B}\left(I\cap {\tilde{L}}_{k+1}\right)$$

(21) $$w_{S}^{\left(I\right)}\left(L\right)={\left[\eta_{S}^{\left(I\right)}\right]}^{L}\sum_{J\in {\tilde{L}}_{0:k}}1_{J}\left(L\right){\left[1-\eta_{S}^{\left(I\right)}\right]}^{J-L}w_{k}^{\left(I\right)}\left(J\right)$$

(22) $$p_{k+1|k}^{\left(I\right)}\left(x,l\right)=1_{{\tilde{L}}_{0:k}}\left(l\right)P_{S}^{\left(I\right)}\left(x,l\right)+\left(1-1_{{\tilde{L}}_{0:k}}\left(l\right)\right)p_{B}\left(x,l\right)$$

(23) $$P_{S}^{\left(I\right)}\left(x,l\right)=\frac{\langle p_{S}\left(\cdot ,l\right)f_{k+1|k}\left(x|\cdot ,l\right),p_{k}^{\left(I\right)}\left(\cdot ,l\right)\rangle }{\eta_{S}^{\left(I\right)}\left(l\right)}$$

(24) $$\eta_{S}^{\left(I\right)}\left(l\right)=\langle p_{S}\left(\cdot ,l\right),p_{k}^{\left(I\right)}\left(\cdot ,l\right)\rangle$$

*$f_{k+1|k}\left(\cdot \right)$ denotes a single-target transition function. $p_{S}\left(x,l\right)$ denotes the survival probability of target x. ${\tilde{L}}_{k+1}$ is the label space of a target born at time k+1.*

**Proposition** **2.** *If the multi-target prediction density has the form of δ-GLMB as (19), then, with the measurement set S and separable likelihood function $\gamma_{S}\left(x\right)$, the multi-target posterior density also has the same form [23]:*

(25) $$v_{k+1}\left(X|S\right)=\Delta \left(X\right)\sum_{I\in F({\tilde{L}}_{0:k+1})}\delta_{I}\left(L\left(X\right)\right)w_{k+1}^{\left(I\right)}\left(S\right){\left[p_{k+1}^{\left(I\right)}\left(\cdot |S\right)\right]}^{X}$$

*where*

(26) $$w_{k+1}^{\left(I\right)}\left(S\right)\propto w_{k+1|k}^{\left(I\right)}{\left[\eta_{S}\right]}^{I}$$

(27) pk+1(I)(x,l|S)=pk+1|k(I)(x,l)γS(x,l)pk+1|k(I)(x,l)γS(x,l)ηS(l)ηS(l)

(28) $$\eta_{S}\left(l\right)=\langle p_{k+1|k}^{\left(I\right)}\left(\cdot ,l\right),\gamma_{S}\left(\cdot ,l\right)\rangle .$$

**Proposition** **3.** *If the multi-target state posterior with the δ-GLMB form is given as (15) at time k, the cardinality distribution $\rho_{k}\left(n\right)$ can be given by [20]*

(29) $$\rho_{k}\left(n\right)=\sum_{I\in F({\tilde{L}}_{0:k})}\delta_{n}\left(\left|I\right|\right)w_{k}^{\left(I\right)}.$$

*The cardinality estimates can be obtained by*

(30) $$\hat{n}=\arg\max_{n}\rho_{k}\left(n\right)$$

The multi-target state estimate is the mean estimate of the multi-target state conditioned on the estimated cardinality $\hat{n}$ as in [20].

#### 2.3.2. Recursion

Particle implementation is based on the standard δ-GLMB filter implementation [20,21]. Each target density $p_{k}^{\left(l\right)}$ with label l in $p_{k}^{\left(I\right)}$ is modeled as a set of weighted samples ${\left\{\left(\Omega_{k,n}^{\left(l\right)},x_{k,n}\right)\right\}}_{n=1}^{J_{k}^{\left(l\right)}}$, where $J_{k}^{\left(l\right)}$ is the number of particles, and $x_{n}$ denotes the state $x_{n}$ and label *l*. Besides the prediction and update steps, we add four other parts: pruning, splitting, merging, and new birth target initialization to form the whole solution. The details are as follows.

(1) Prediction 

The pixels illuminated by the target can describe the target more effectively than a single point or a rectangle. Let us assume the single-target state *x* have two fields: a geometric center location x.cen and cover area x.cov, which can be obtained from T(x). For convenience, let x.cen=x.cen and x.cov=x.cov in this paper. This new approach representing a single-target benefits from the similar target shape in consecutive frames. To accommodate the small amount of deformation, pixel sampling is executed in this prediction step. Each pixel in x.cov has the probability of $p_{pix}$ to be selected to survive, the value of which depends on the deformation degree. In general, when the deformation is small, $p_{pix}$ is close to 1. It is obvious that sampling may reduce the covered area in the final estimates. Fortunately, this can be alleviated in the update step.

If at time *k*, the multi-target state posterior is given by (15), when the LMB new birth model ${\left\{\left(r_{B,k+1}^{\left(l\right)},p_{B,k+1}^{\left(l\right)}\right)\right\}}_{l\in {\tilde{L}}_{k+1}}$ is known (in practice for the unknown birth model, the third part in this subsection describes how to initialize the new birth model), where $p_{B,k+1}^{\left(l\right)}$ is ${\left\{\left(\Omega_{B,k+1,n}^{\left(l\right)},x_{B,k+1,n}\right)\right\}}_{n=1}^{B^{\left(l\right)}}$ and $B^{\left(l\right)}$ is the number of particles, according to proposition 1, we obtain

(31) $$\eta_{S}^{\left(I\right)}\left(l\right)=\sum_{n=1}^{J_{k}^{\left(l\right)}}\Omega_{k,n}^{\left(l\right)}p_{S}\left(x_{k,n}\right)$$

and $p_{k+1|k}^{\left(I\right)}\left(x,l\right)$ can be represented as

(32) $${\left\{\left(1_{{\tilde{L}}_{0:k}}\left(l\right){\tilde{\Omega }}_{S,k+1|k,n}^{\left(l\right)},x_{S,k+1|k,n}\right)\right\}}_{i=n}^{J_{k}^{\left(l\right)}}\cup {\left\{\left(1_{{\tilde{L}}_{0:k}}\left(l\right)\Omega_{B,k+1,n}^{\left(l\right)},x_{B,k+1,n}\right)\right\}}_{i=n}^{B^{\left(l\right)}},$$

where $x_{S,k+1|k,n}.cen∼q\left(\cdot |x_{k,n}.cen\right)$ ($n=1,2,...,J_{k}^{\left(l\right)}$), $x_{S,k+1|k,n}.cov$ is generated by a random pixel sample as described above; $\Omega_{S,k+1|k,n}^{\left(l\right)}=\frac{\Omega_{k,n}^{\left(l\right)}f_{k+1|k}\left(x_{S,k+1|k,n}|x_{k,n},l\right)p_{S}\left(x_{k,n},l\right)}{q(x_{S,k+1|k,n}.cen|x_{k,n}.cen)}$; Ω˜S,k+1|k,n(l)=ΩS,k+1|k,n(l)ΩS,k+1|k,n(l)∑n=1Jk(l)ΩS,k+1|k,n(l)/∑n=1Jk(l)ΩS,k+1|k,n(l); and $q(\cdot |x_{k,n}.cen)$ is a proposal density. The procedure for calculating $w_{k+1|k}^{\left(I\right)}$ in (20) is bothersome and complex; however, reference [21] offers a method based on a K-shortest paths algorithm to carry it out. To understand the procedure more clearly, reference [21] is strongly recommended.

(2) Update 

With the assumption that targets are not overlapped, we obtain a separable likelihood function shown as (12). After adding a distinct label to each target, (12) can be written as

(33) $$g\left(S\left(k\right)|X\right)\propto \prod_{x\in X}\gamma_{S}\left(x\right)$$

where $\gamma_{S}\left(x\right)=g_{F}\left(\bar{s}\left(x\right)\right)$.

According to proposition 2, if each single-target density $p_{k+1|k}^{\left(l\right)}$ is modeled by a particle set ${\left\{\left(\Omega_{k+1|k,n}^{\left(l\right)},x_{k+1|k,n}\right)\right\}}_{n=1}^{J_{k+1|k}^{\left(l\right)}}$. Then,

(34) $$\eta_{S}\left(l\right)=\sum_{n=1}^{J_{k+1|k}\left(l\right)}w_{k+1|k,n}\left(l\right)\gamma_{S}\left(x_{k+1|k,n}\right)$$

and $p_{k+1}^{\left(I\right)}\left(x,l|S\right)$ can be represented as

(35) $${\left\{\left(\Omega_{k+1,n}^{\left(l\right)},x_{k+1,n}\right)\right\}}_{n=1}^{J_{k+1}^{\left(l\right)}},$$

where $w_{k+1,n}\left(l\right)=w_{k+1|k,n}\left(l\right)\gamma_{S}\left(x_{k+1|k,n}\right)/\sum_{n=1}^{J_{k+1|k}\left(l\right)}w_{k+1|k,n}\left(l\right)\gamma_{S}\left(x_{k+1|k,n}\right)\;$; $x_{k+1,n}=x_{k+1|k,n}$; and $J_{k+1}^{\left(l\right)}=J_{k+1|k}^{\left(l\right)}$. Substituting (34) into (26), we obtain $w_{k+1}^{\left(I\right)}\left(S\right)$.

Analogous to the standard particle filter, resampling each target density ${\left\{\left(\Omega_{k+1,n}^{\left(l\right)},x_{k+1,n}\right)\right\}}_{n=1}^{J_{k+1}^{\left(l\right)}}$ must be executed to reduce the degeneracy. For simplicity, in this paper, multinomial resampling is used for numerical studies that would otherwise be carried out with other multi-Bernoulli filters [17].

To eliminate the influence of pixel sampling in the prediction step, a pixel set update procedure is used to correct the shape of the target. Taking target *x* as an example, the procedure is described as follows. First, for all $J_{k+1}^{\left(l\right)}$ particles representing target *x*, count the pixel *i* ($i\in {⋃}_{j=1}^{J_{k+1}^{\left(l\right)}}T({x_{k+1}}_{,j})$) occupied times $OT_{i}^{J_{k+1}^{\left(l\right)}}$. The occupied times are initialized to 0, i.e., $OT_{i}^{0}=0$, and for the jth ($1\leq j\leq J_{k+1}^{\left(l\right)}$) particle state ${x_{k+1}}_{,j}$, $OT_{i}^{j}$ is given by

(36) $$OT_{i}^{j}=\left\{\begin{array}{cc} OT_{i}^{j-1}+1 & if\;i\in T({x_{k+1}}_{,j})\\ OT_{i}^{j-1} & otherwise \end{array}\right..$$

Second, compare the $OT_{i}^{J_{k+1}^{\left(l\right)}}$ with a threshold ppixJk+1(l)ppixJk+1(l)22. When $OT_{i}^{J_{k+1}^{\left(l\right)}}$ reaches the threshold, the pixel *i* is classified as an occupied pixel. Third, if the occupied pixel *i* is greater than an extremely low value in the foreground probability map, then it is considered to be a pixel in the updated pixel set for target *x*. The updated pixel set is the estimated cover area of target *x*. The pseudo-code for the pixel set update procedure is presented in Algorithm 2. Algorithm 2 shows us that for each pixel belonging to the covered areas occupied by all particles representing *x*, the occupied time is calculated (line 3) and then it will be used to determine the true cover area of the target (lines 5–9). The filter will propagate a pixel set as the target state in the recursion and will directly produce the pixel set as its output. This operation can facilitate target recognition and extraction and subsequent processing in computer vision applications.

**Algorithm 2:** Pixel set update

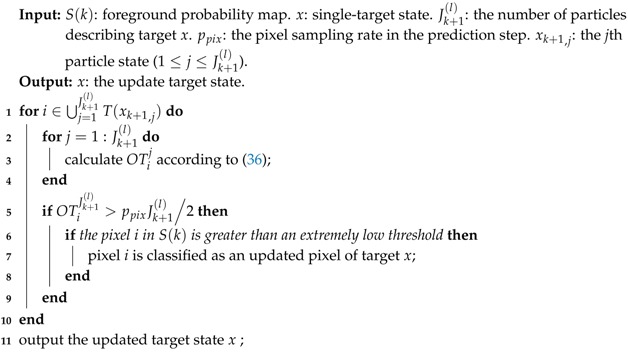



(3) Pruning, Splitting, Merging and Birth Target Initialization 

To reduce computational complexity, besides the truncation [20,21], the pruning procedure is also needed. The multi-target set X should be discarded if its weight $w_{k+1}^{\left(I\right)}$ is less than a threshold $th_{pr}$. In practical application, the dynamic processes of multi-targets are complex. To accommodate these processes, we propose splitting and merging, which are described as follows. For each target x, clustering is executed to check whether the target x should be split into several small targets. If yes, the small target with the most pixels inherits the identity (label) of target x, while the others are labeled as new birth targets. For two targets in X with substantial overlap, if the overlap ratio of the pixel intersection area to the smaller target area is higher than a threshold $th_{me}$, these two targets should be merged; the merged label is the same as the earlier born target.

Birth target initialization has two pre-processing steps. Step 1: clustering the foreground probability map to obtain current cluster target $x_{c,i}$ (0≤i≤n, *n* denotes the number of all current cluster targets). Step 2: for each cluster target $x_{c,i}$, calculate the overlap ratio between the $x_{c,i}$ and all existing targets. If the maximum overlap ratio is higher than a threshold $th_{bir}$, then remove this $x_{c,i}$. Subsequently, we use the remaining $x_{c,i}$ to initiate the new birth target LMB modeled as $v_{B,k+1}={\left\{\left(r_{B,k+1}^{\left(l\right)},p_{B,k+1}^{\left(l\right)}\right)\right\}}_{l\in {\tilde{L}}_{k+1}}$. For simplicity, the $r_{B,k+1}^{\left(l\right)}$ is initialized as a constant and the target density $p_{B,k+1}^{\left(l\right)}$ is modeled as ${\left\{\left(\Omega_{B,k+1,n}^{\left(l\right)},x_{B,k+1,n}\right)\right\}}_{n=1}^{B^{\left(l\right)}}$, where all $\Omega_{B,k+1,n}^{\left(l\right)}$ have equal weight; and $x_{B,k+1,n}.cen∼N\left(u_{B}^{\left(l\right)},Q_{B}^{\left(l\right)}\right)$, where N(·) denotes the Gaussian distribution, $u_{B}^{\left(l\right)}$ is the geometric center location of one remaining $x_{c,i}$, and $Q_{B}^{\left(l\right)}$ is the variance. $x_{B,k+1,n}.\mathrm{cov}$ can be obtained from $T(x_{c,i})$ using random sampling by probability $p_{pix}$.

These procedures are designed to broaden the applications of the filter. Without splitting and merging, this filter would produce a cardinality estimation error. Missing the new target birth process would invalidate the δ-GLMB-TBD filter. Using proposition 3 can produce a multi-target estimate.

## 3. Experimental Results

In this section, the optimal parameters of the background-subtraction-based δ-GLMB-TBD filter are determined. Based on the optimal parameters, detection performance is compared to several state-of-the-art techniques. Then, tracking performance is tested. Based on experimental results, the advantages and disadvantages of the proposed algorithm are discussed. In these experiments, the kinematic transition is modeled as the random walk model; the survival probability of a target is 0.98; the maximum number of frames in which the target remains stationary $N_{sta}$ is 60; the morphology erosion and dilation operators are executed once with the same flat disk-shaped structuring element whose radius is 1. The particle number for each target is 200; the pruning threshold $th_{pr}$ is 0.01; the DBSCAN method is chosen for clustering [32]; the threshold $th_{bir}$ in birth targets initialization is 0.5; the merging threshold $th_{me}$ is 0.7; the existence probability $r_{B,k+1}^{\left(l\right)}$ is 0.3; and the variance $Q_{B}^{\left(l\right)}$ is [1,0;0,1]. Theoretically, the same detection result and data association can achieve the same tracking performance. Therefore, the detection performance is the main evaluation criteria for the joint detection and tracking method. This novel TBD filter emphasizes detection by exploiting the trajectory information. The measure metrics of recall, precision, and F-measure (FM) are used to evaluate performance, and they are defined as:

(37) $$recall=\frac{tp}{tp+fn},precision=\frac{tp}{tp+fp},FM=\frac{2\cdot precision\cdot recall}{precision+recall}$$

where tp denotes the number of true positives, and fp and fn represent the number of false positives and false negatives.

### 3.1. Determination of Parameters

From previous discussions, the parameters to be determined are as follows: (1) the threshold $th_{diff}$ in (4), (2) the number of samples *N*, (3) $\delta_{F}$ and $\delta_{B}$, and (4) pixel sampling rate $p_{pix}$. The tested sequence is OTCBVS Dataset 03 2a [33]. The fluctuation of the $\delta_{F}$ to $\delta_{B}$ ratio does not cause obvious changes in the tracking result, where $\delta_{F}=0.2$ and $\delta_{B}=0.05$ are suitable for our experiments.

The proposed method can be divided into two parts: detection and tracking, which are connected by the foreground probability map S(k). The two parts can be considered as conditional independence (CI) based on S(k). Therefore, the parameters also can be divided into two groups: (1) $th_{diff}$ and *N* (before obtaining S(k)); (2) $p_{pix}$ (after obtaining S(k)). Let us use the “trial-and-error” method to determine the two groups of parameters respectively as follows.

To choose the optimal value for $th_{diff}$ and *N*, the performance metrics are calculated for $th_{diff}$ as: 20, 40, 60, 80, and 100 while *N* as: 10, 20, 30, 40, and 50. The other parameters are fixed at $\delta_{F}$ = 0.2, $\delta_{B}$ = 0.05 and $p_{pix}$ = 0.8. The results are shown in Table 1, Table 2 and Table 3. From Table 1, we can see that the recall decreases with increasing $th_{diff}$. When $th_{diff}$ is set 20, the recall can achieve the highest value. The number of samples *N* has less influence on recall; in general, with the increase of *N*, the recall tends to become higher. From Table 2, the result of precision is opposite to the recall metric. Table 3 shows that the balance metric FM has the highest value at $th_{diff}$ = 60. In this TBD method, recall plays a more important role than precision. The range of $th_{diff}$ can be set between 40 and 60, *N* is between 20 and 40.

Now, let us determine the pixel sampling rate $p_{pix}$. When we set $th_{diff}$ to 60, $\delta_{F}$ to 0.2, $\delta_{B}$ to 0.05, and *N* to 20, Table 4 shows the experimental results. From Table 4, the evaluation metrics do not change significantly, because the $p_{pix}$ mainly affects the detection and tracking of dim targets that occupy few pixels and may be deformed in consecutive frames. A $p_{pix}$ closer to 1 is of minimal help in detecting and tracking deformation targets, and a smaller $p_{pix}$ may cause a dim target to be undetected. In the experiment, the $p_{pix}$ is set to 0.8.

### 3.2. Comparison with Other Techniques

To the best of our knowledge, there is no common dataset for TIR multiple moving-target detection and tracking in the surveillance scene. We collected 15 sequences containing about 14,753 images in 7 different locations at different times from OTCBVS [34] (IEEE OTCBVS WS Series Bench and Roland Miezianko, Terravic Reasearch Infrared Database) and PTB-TIR [35]. The moving targets are mainly pedestrians, but also include pets, cars, and trucks. In this subsection, the performance of the proposed method is compared with 6 methods: ViBe [24], pixel-based adaptive segmenter (PBAS) [36], and kernel density estimator (KDE) [25], SLIC-based method [37], orthogonal rank-one matrix pursuit method (OR1MP) [38], and robust PCA via Gradient Descent (RPCA-GD) [39]. The ViBe, PBAS, and KDE are classic state-of-the-art methods. The SLIC, OR1MP, and RPCA-GD are 3 new methods. Among them, SLIC is a proposal method designed for infrared target detection; OR1MP and RPCA-GD can be recursive and unable to give instantaneous detection because of the non-causality. The programs of the ViBe, KDE, OR1MP, and RPCA-GD methods are provided by their authors, and the parameters used in the experiments are suggested by the authors. The PBAS method is available in the BGSlibrary [40]. SLIC is implemented by us using MATLAB; we define $th_{d}=means+4stds$ as the detection threshold, where means and stds are the mean and the standard deviation of all saliency scores, respectively.

First, we use four typical sequences to validate the effectiveness of the proposed method. They cover many typical situations such as targets crossing, entering/leaving, gathering/separation, occlusion, stopping /restarting, and irregular motion. The sequences are divided into two categories: sparse target scenarios and dense target scenarios. The results of the four sequences are well represented for examining the performance of the proposed method. The parameters used in our model are $th_{diff}$ = 60, N=20, $\delta_{F}=0.2$, $\delta_{B}=0.05$, and $p_{pix}=0.8$. For the proposed method, the output results are the estimates of the filter, while for other methods, the results are the foreground detections.

**Figure 4:**
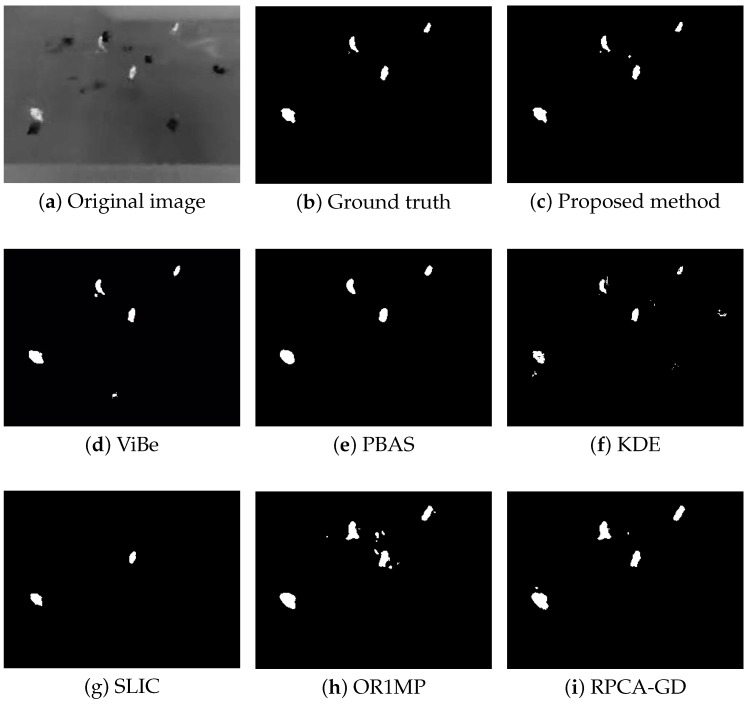
The detection results of the 190th frame in Seq 1.

**Figure 5:**
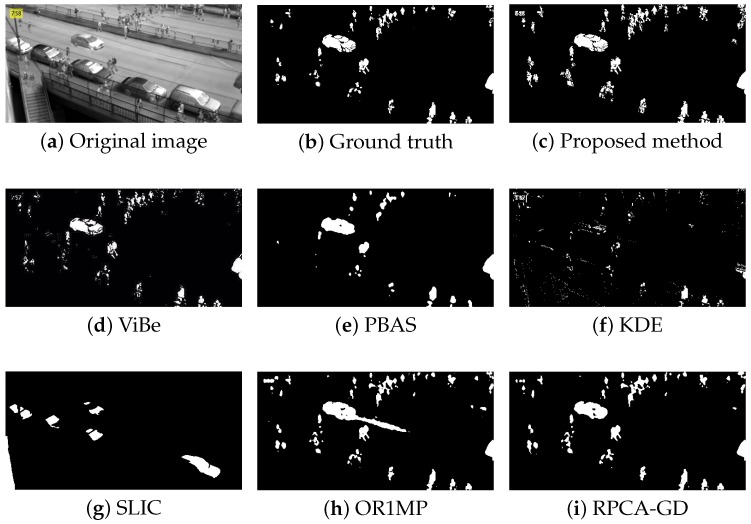
The detection results of the 758th frame in Seq. 2.

**Figure 6:**
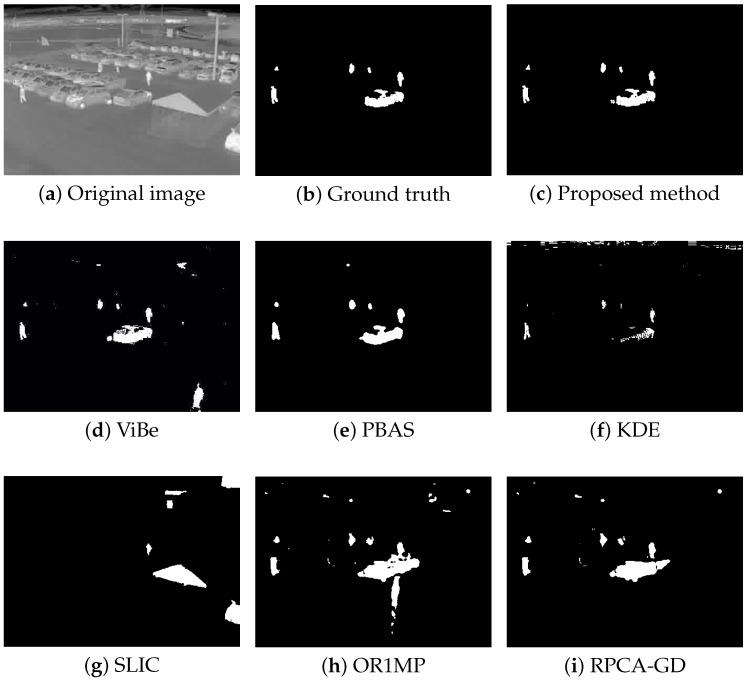
The detection results of the 173th frame in Seq. 3.

**Figure 7:**
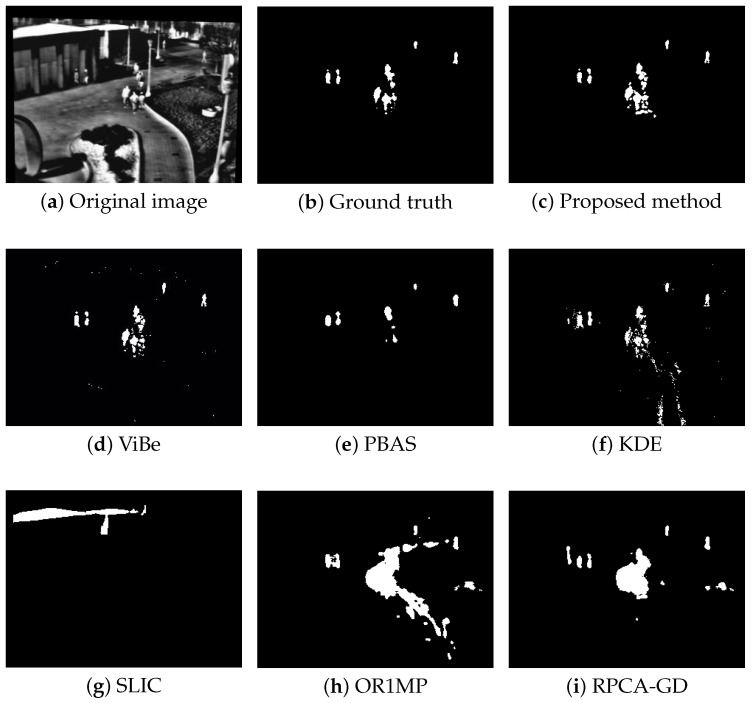
The detection results of the 741rd frame in Seq. 4.

Figure 4, Figure 5, Figure 6 and Figure 7 shows examples of moving-target detection for four typical frames chosen from four different sequences. Table 5 presents the average evaluation metrics of the seven methods. For all four sequences, the proposed method can detect all moving targets, despite some false alarms. Compared with our method, ViBe detects more false alarms and PBAS produces missing detections. The KDE produces obvious worse detection results than the proposed method. Because the proposal-based method only uses the features of the target to detect and pay no attention to the information in inter frame, SLIC can only detect the highlight areas in the images. In general, other algorithms that do not use information between consecutive frames, such as the SLIC method, will obtain similar results. OR1MP and RPCA-GD can also obtain good detection performance on Seq. 2; this is because their non-causality allows them to use the follow-up frames to build the current time model to eliminate the ghost. The non-causality will limit their application. However, for Seq.1, OR1MP and RPCA-GD detection results occupy more pixels than the ground truth; for Seq. 3 and Seq. 4 they produce obvious missing detection. These results are validated by the metric scores in Table 5. The good performance of the proposed method benefits from the detection support map, the neighbor pixel update processing, and the use of trajectory information. Figure 4, Figure 5, Figure 6 and Figure 7 and Table 5 indicate that the proposed method outperforms the other six methods obviously.

In addition, Table 6 shows average evaluation metrics and runtime per frame for all 15 sequences. From Table 6, we can see that the proposed method can get the highest recall, precision, and FM metrics even compared with the non-causal method. Also, Table 6 validates that the proposed method has the best detection performance among the 7 methods.

### 3.3. Discussion of Tracking Performance

The background-subtraction-based δ-GLMB-TBD method can also produce target trajectories without additional post-processing, and accommodate multi-target dynamic process and measurement process, although its main task is target detection. In this section, the optimal sub-pattern assignment (OSPA) metric [41] interpreted as per-target tracking error containing cardinality error and state estimation error with parameters p = 1 and c = 50 pixel will be employed as the main performance metric.

**Figure 8:**
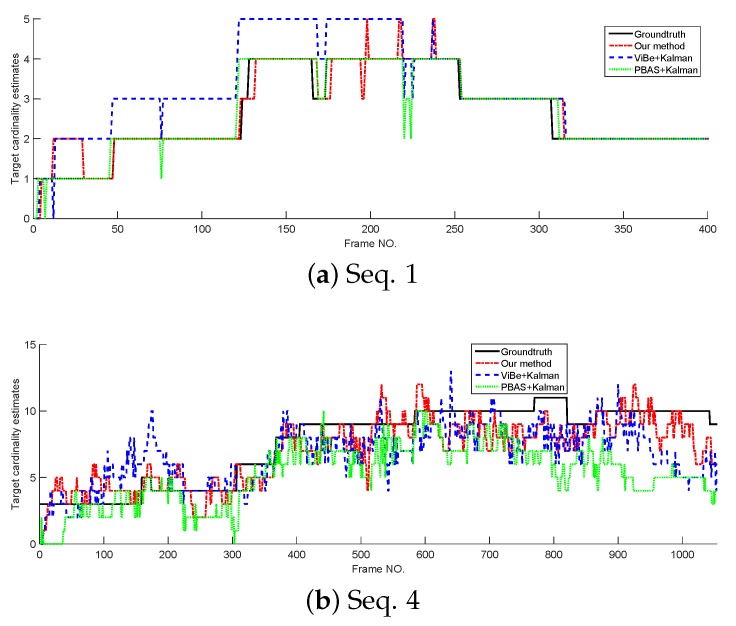
The cardinality estimates of different trackers.

**Figure 9:**
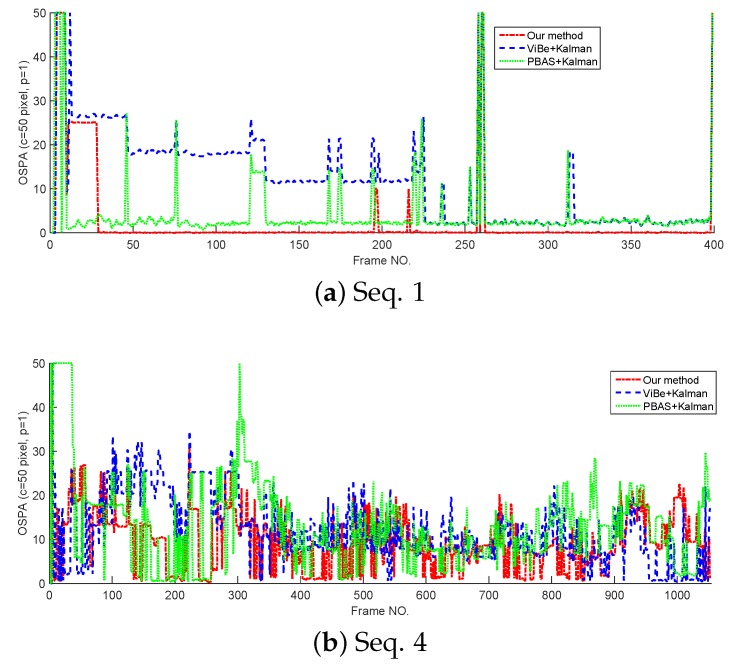
The OSPA of different trackers.

In this section, the tracking performance of the proposed method is compared with the standard Kalman filter whose detections are provided by ViBe and PBAS methods. The models and parameters used in Kalman filter are the same as the proposed method, but in Kalman filters, the target state is represented by its centroid. The cardinality estimates and OSPA curves tested on Seq. 1 and Seq. 4 are shown in Figure 8 and Figure 9. From Figure 8; we can see that without overlapped targets, our method and PBAS + Kalman method can almost produce the correct cardinality; and with complex multi-target motion and occlusion, the 3 filters produce obvious cardinality estimation error, but they still can reflect the trend of cardinality change. From Figure 9, we can see, in both simple and complex scenarios, the proposed method can obtain the lowest OSPA value, this means the proposed method has the best multi-target tracking performance in the 3 methods. This is because the proposed method can yield accurate appearance and centroid estimation. The average OSPA values of all 15 sequences is shown in Table 7. Also, Table 7 indicates the proposed method can obtain the best multi-target tracking performance.

**Figure 10:**
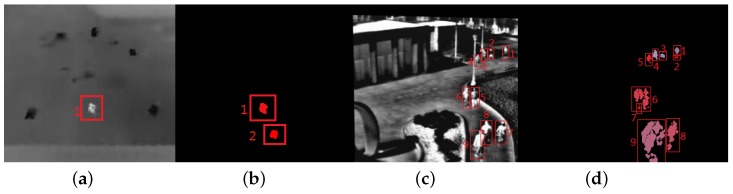
The two typical results of the proposed method. (**a**) The 20th frame in Seq. 1; (**b**) the proposed method estimates of (**a**); (**c**) the 486th frame in Seq. 4; (**b**) the proposed method estimates of (**c**).

Figure 10 shows two typical frames with estimation error. The proposed method outputs targets that are filled with different colors. From Figure 10, we can see the “ghost” (seen in Figure 10b), similarity between the targets and background (red rectangles with number 1, 2, 6, 7 in Figure 10b) can cause the over estimation; occlusions between targets or between target and background (red rectangles with number 6, 9 in Figure 10b) is the main reason to produce low estimation.

**Figure 11:**
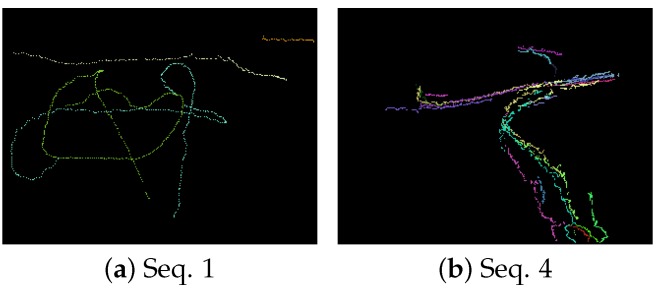
The trajectory estimate results.

As described above, this new filter can add a label to the target to maintain the track. Figure 11 shows the estimated trajectories of moving targets whose duration exceeds 30 frames. The different colors in Figure 11 denote different trajectories. According to Figure 11, the filter can track the targets successfully along with their labels without overlap or occlusion. When a target is separated from the crowd, this new filter can continue tracking it as a new target. The estimates from the labeled filter can facilitate tracking, recognition, and other subsequent processing in computer vision. In future, the trace association could be used to merge the tracks before and after occlusion to form a long-time trajectory.

## 4. Conclusions

A novel method for moving-target detection and tracking directly from the TIR sequence in surveillance scenes was proposed based on background-subtraction and the δ-GLMB-TBD filter. First, a background subtraction method using a random selection strategy was used to produce the foreground probability map. Separable non-overlapped multi-target likelihood was exploited to obtain the probability of the pixels belonging to the foreground. Then, the δ-GLMB-TBD filter was used to provide estimates. Unlike other RFS-based filters, the proposed method used the pixel set, which was the target projection in the image, to describe the target instead of a rectangle or a single point. This means the δ-GLMB-TBD filter directly produced a continuous trajectory as well as accurate multi-target shape estimates. In implementation, several procedures including pixel sampling and update, target merging and splitting, and new birth target initialization were combined in the method to accommodate target deformation and multi-target dynamic change: gathering, splitting, birth, and death. After describing the method, the optimal parameters were determined by experiments. Then, the performance of the novel method was compared with six existing methods. According to the experimental results, the proposed TBD method obtained the highest FM scores, meaning that it outperformed the other six methods in the detection of moving targets. The experiments also show that the proposed method can achieve better tracking performance than the Kalman filters with different detections. In future, the proposed method could be extended to the detection and tracking of moving targets without non-overlap assumptions. 

References

References

## Figures and Tables

**Table 1:** Average recall with different $th_{diff}$ and *N*.

	*N*	10	20	30	40	50
${\mathrm{th}}_{\mathrm{diff}}$						
20		0.9315	0.9219	0.9057	0.9082	0.9241
40		0.8560	0.8532	0.8549	0.8756	0.8654
60		0.7311	0.7880	0.8031	0.8121	0.8213
80		0.6343	0.6996	0.7146	0.7108	0.7288
100		0.5083	0.5804	0.6307	0.6316	0.6423

**Table 2:** Average precision with different $th_{diff}$ and *N*.

	*N*	10	20	30	40	50
${\mathrm{th}}_{\mathrm{diff}}$						
20		0.4710	0.3937	0.3557	0.3361	0.3158
40		0.7279	0.6752	0.6461	0.6206	0.6039
60		0.8311	0.8296	0.8146	0.8056	0.8049
80		0.8910	0.8949	0.8931	0.8889	0.8853
100		0.9316	0.9304	0.9374	0.9349	0.9338

**Table 3:** Average FM with different $th_{diff}$ and *N*.

	*N*	10	20	30	40	50
${\mathrm{th}}_{\mathrm{diff}}$						
20		0.6221	0.5482	0.5061	0.4847	0.4665
40		0.7848	0.7479	0.7303	0.7228	0.7077
60		0.7709	0.8052	0.8050	0.8048	0.8031
80		0.7358	0.7796	0.7909	0.7849	0.7964
100		0.6480	0.7062	0.7498	0.7477	0.7552

**Table 4:** Average evaluation metrics of different $p_{pix}$.

$p_{\mathrm{pix}}$	0.6	0.7	0.8	0.9	1
recall	0.9204	0.9157	0.9234	0.9296	0.9299
precision	0.8085	0.8047	0.8020	0.8037	0.8020
FM	0.8532	0.8431	0.8510	0.8545	0.8539

**Table 5:** Average evaluation metrics of different methods.

Metrics		Proposed Method	ViBe	PBAS	KDE	SLIC	OR1MP	RPCA-GD
Seq. 1	recall	0.9996	0.9875	0.9434	0.8477	0.6090	0.9992	0.9996
	precision	0.9487	0.8489	0.7743	0.8266	0.8411	0.5716	0.6175
	FM	0.9587	0.8952	0.8269	0.8295	0.6216	0.7169	0.7533
Seq. 2	recall	0.9625	0.7127	0.6132	0.3660	0.0679	0.9598	0.9621
	precision	0.8231	0.8487	0.8523	0.7512	0.0633	0.67851	0.8031
	FM	0.8663	0.7893	0.7150	0.4547	0.0611	0.8076	0.8651
Seq. 3	recall	0.9656	0.9632	0.9007	0.5276	0.3869	0.5238	0.5778
	precision	0.8477	0.4931	0.6529	0.5408	0.1362	0.7826	0.7743
	FM	0.8764	0.6240	0.7096	0.5107	0.1874	0.5618	0.6020
Seq. 4	recall	0.8847	0.8311	0.6150	0.7524	0.0210	0.7477	0.7786
	precision	0.7909	0.7142	0.8731	0.6306	0.0257	0.8103	0.7776
	FM	0.8244	0.7626	0.7035	0.6784	0.0173	0.7716	0.7669

**Table 6:** Average evaluation metrics for all sequences.

	Proposed Method	ViBe	PBAS	KDE	SLIC	OR1MP	RPCA-GD
recall	0.9839	0.9332	0.8619	0.7682	0.4435	0.8449	0.8714
precision	0.8732	0.6116	0.8121	0.5993	0.3082	0.7871	0.7935
FM	0.9094	0.6438	0.8001	0.5600	0.1441	0.7543	0.7775

**Table 7:** Average OSPA values for all sequences.

Proposed Method	ViBe + Kalman	PBAS + Kalman
5.00	11.13	9.16
